# The Anti-Inflammatory Effect of Acidic Mammalian Chitinase Inhibitor OAT-177 in DSS-Induced Mouse Model of Colitis

**DOI:** 10.3390/ijms23042159

**Published:** 2022-02-15

**Authors:** Marzena Mazur, Jakub Włodarczyk, Mikołaj Świerczyński, Radzisław Kordek, Marcin M. Grzybowski, Jacek Olczak, Jakub Fichna

**Affiliations:** 1 Department of Biochemistry, Faculty of Medicine, Medical University of Lodz, Mazowiecka 6/8, 92-215 Lodz, Poland; m.mazur@oncoarendi.com (M.M.); jakub.wlodarczyk@stud.umed.lodz.pl (J.W.); mikolaj.swierczynski@stud.umed.lodz.pl (M.Ś.); 2 OncoArendi Therapeutics SA, Żwirki i Wigury 101, 02-089 Warsaw, Poland; m.grzybowski@oncoarendi.com (M.M.G.); j.olczak@oncoarendi.com (J.O.); 3 Department of Pathology, Faculty of Medicine, Medical University of Lodz, Pomorska 251, 92-213 Lodz, Poland; radzislaw.kordek@umed.lodz.pl

**Keywords:** acidic mammalian chitinase, colitis, inflammatory bowel diseases, inhibitor

## Abstract

Inflammatory bowel diseases (IBD) are chronic and relapsing gastrointestinal disorders, where a significant proportion of patients are unresponsive or lose response to traditional and currently used therapies. In the current study, we propose a new concept for anti-inflammatory treatment based on a selective acidic mammalian chitinase (AMCase) inhibitor. The functions of chitinases remain unclear, but they have been shown to be implicated in the pathology of various inflammatory disorders regarding the lung (asthma, idiopathic pulmonary fibrosis) and gastrointestinal tract (IBD and colon cancer). The aim of the study is to investigate the impact of AMCase inhibitor (OAT-177) on the dextran sulfate sodium (DSS)-induced models of colitis. In the short-term therapeutic protocol, OAT-177 given intragastrically in a 30 mg/kg dose, twice daily, produced a significant (*p* < 0.001) anti-inflammatory effect, as shown by the macroscopic score. Additionally, OAT-177 significantly decreased TNF-α mRNA levels and MPO activity compared to DSS-only treated mice. Intraperitoneal administration of OAT-177 at a dose of 50 mg/kg caused statistically relevant reduction of the colon length. In the long-term therapeutic protocol, OAT-177 given intragastrically in a dose of 30 mg/kg, twice daily, significantly improved colon length and body weight compared to DSS-induced colitis. This is the first study proving that AMCase inhibitors may have therapeutic potential in the treatment of IBD.

## 1. Introduction

Inflammatory bowel diseases (IBD), including ulcerative colitis (UC) and Crohn’s disease (CD), are chronic and relapsing intestinal disorders characterized by prolonged inflammation of the digestive tract. The precise etiology of IBD remains unknown despite ongoing studies. It is believed that besides the genetic and environmental factors, a dysregulated immune system, intestinal microbiota, and dysfunction in the mucosal barrier are also involved [1,2]. Treatment strategies currently used in IBD rely mainly on reduction of inflammation by administration of aminosalicylates, corticosteroids, and immunomodulators, or inhibition of TNFα [3]. However, a significant proportion of patients are unresponsive or lose response to these traditional therapies. What is more, current IBD treatments are associated with significant side effects, including neoplasia [4]. There are a number of promising clinical trials ongoing—for example, those based on α4β7 and α4β1 integrin inhibitors and IL-12/IL-23 inhibitors [5,6]. Stem-cell transplant and fecal microbiota transplant also show a beneficial effect as experimental therapies [7,8]. However, it is still necessary to develop alternative and novel approaches in IBD treatment based on new molecular targets.

Two chitinases, chitotriosidase-1 (CHIT-1) and acidic mammalian chitinase (AMCase)—enzymes belonging to the GH 18 family that catalyze cleavage of 1,4-b-glycosidic bond in chitin and chitooligosaccharides—have been identified as factors regulating the GI tract functioning [9]. CHIT-1, expressed by activated macrophages, appears to be an important component in colon homeostasis. It has been found that CHIT1 was down-regulated in active UC patients, while in healthy controls it was actively secreted by mucin-producing cells, and it has been postulated that decreased secretion of this enzyme exacerbates colonic inflammation [10].

While CHIT-1 is produced in a number of organs, AMCase, expressed by the epithelial cells and macrophages, is present mainly in the GI tract and in the lung [11]. It is believed to be responsible for digestion of the food-derived chitin and for the host defense against chitin-producing pathogens present in the GI tract. It is highly upregulated in the pathological conditions involving Th2 inflammatory response, both in animal models of asthma and in allergic patients [12,13].

Several natural products are known to inhibit AMCase, but they are characterized by moderate activity and lack of selectivity versus CHIT-1 [14]. A small-molecule compound OAT-177 [15] has been reported to effectively inhibit murine acidic chitinase (mAMCase IC_50_ = 19 nM), while being much less active versus its sister-enzyme mCHIT-1 (IC_50_ = 3 µM; 155-fold selectivity) and completely inactive against chitinase-like proteins YKL-40 and YKL-39 [16]. OAT-177 showed anti-inflammatory efficacy (evidenced by significant reduction of inflammatory markers) in the asthma animal model driven by the acute HDM-induced allergic airway inflammation [15]. As it is postulated that the tissue remodeling observed in IBD is caused by a similar inflammatory mechanism to the one operating in asthma, we decided to investigate how the selective mAMCase inhibition would impact the progression of the disease in the dextran sulfate sodium (DSS)-induced mouse models of colitis.

## 2. Results

### 2.1. OAT-177 Administered Intragastrically in the Short-Term Therapeutic Protocol Attenuated Colitis in the DSS-Induced Mouse Model

**Figure 1:**
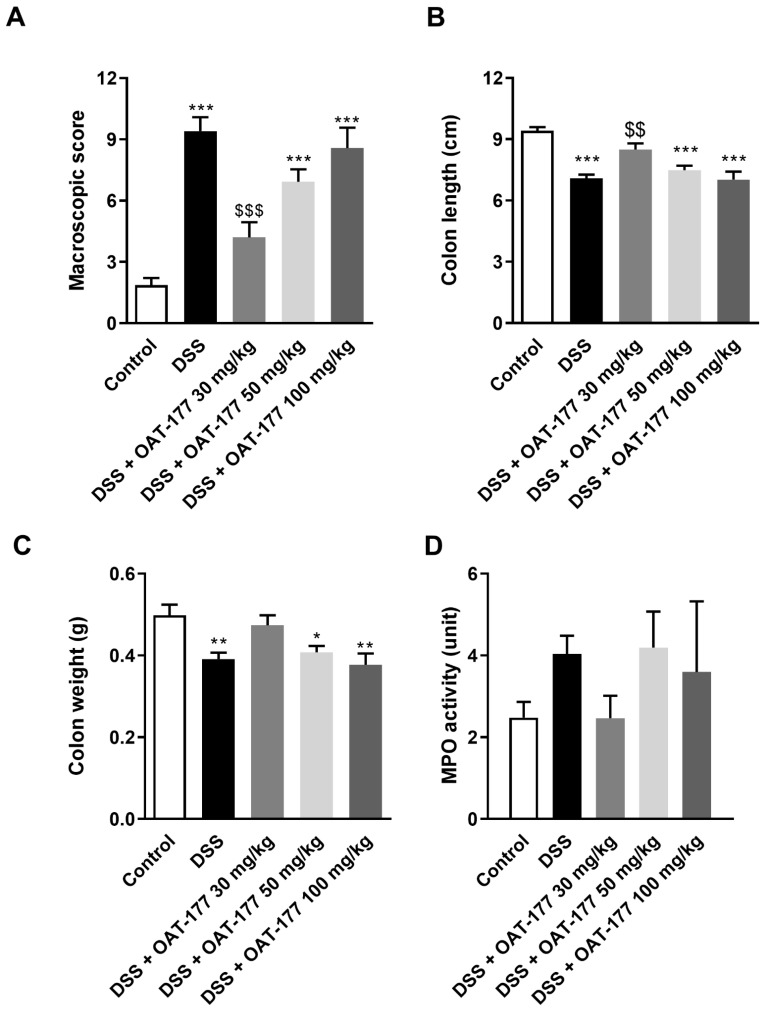
The effect of OAT-177 administered intragastrically in the short-term therapeutic protocol on: (**A**) macroscopic score, (**B**) colon length, (**C**) colon weight, and (**D**) myeloperoxidase (MPO) activity in a dextran sulfate sodium (DSS)-induced colitis. Data are expressed as mean ± SEM. Statistical significance from the one-way ANOVA, Tukey’s post hoc test: *** *p* < 0.001, ** *p* < 0.01, * *p* < 0.05 vs. control group; and $$$ *p* < 0.001, $$ *p* < 0.01 vs. DSS-only treated group.

DSS treatment induced colitis, as evidenced by the increased macroscopic score (9.40 ± 0.69 for DSS-only treated mice vs. 1.87 ± 0.35 for control animals) (Figure 1A), which is consistent with our previous observations [17]. The p.o. administration of OAT-177 in DSS-treated animals decreased the macroscopic score: 4.2 ± 0.74 for a dose of 30 mg/kg, 6.92 ± 0.61 for a dose of 50 mg/kg, and 8.57 ± 1.00 for a dose of 100 mg/kg compared to DSS-only treated mice (respectively, *p* < 0.001; *p* = 0.05; *p* = 0.92) (Figure 1A). Similarly, colon length was decreased in DSS-only treated mice (7.08 ± 0.19 cm vs. 9.42 ± 0.17 cm for control animals), and this effect was reversed by treatment with 30 mg/kg OAT-177 (8.49 ± 0.30 cm, *p* < 0.01) (Figure 1B). Additionally, colon weight was decreased for DSS-only treated mice (0.39 ± 0.01 g vs. 0.50 ± 0.03 g for control animals), while mice treated with the lowest dose of OAT-177 showed colon weight comparable to the control group (0.47 ± 0.02 g) (Figure 1C).

The MPO activity represents neutrophil infiltration of the intestine; therefore, it is the highest in the inflamed tissue [18]. In line, the MPO activity was lowest in the colon of control animals (2.48 ± 0.38 U) and DSS-treated mice, which received OAT-177 at the lowest dose (2.46 ± 0.55 U). Higher MPO activity was observed in the DSS-only treated mice (4.04 ± 0.45 U) and DSS-treated mice upon administration of OAT-177 at doses of 50 mg/kg (4.19 ± 0.88 U) and 100 mg/kg (3.59 ± 1.73 U). (Figure 1D).

**Figure 2:**
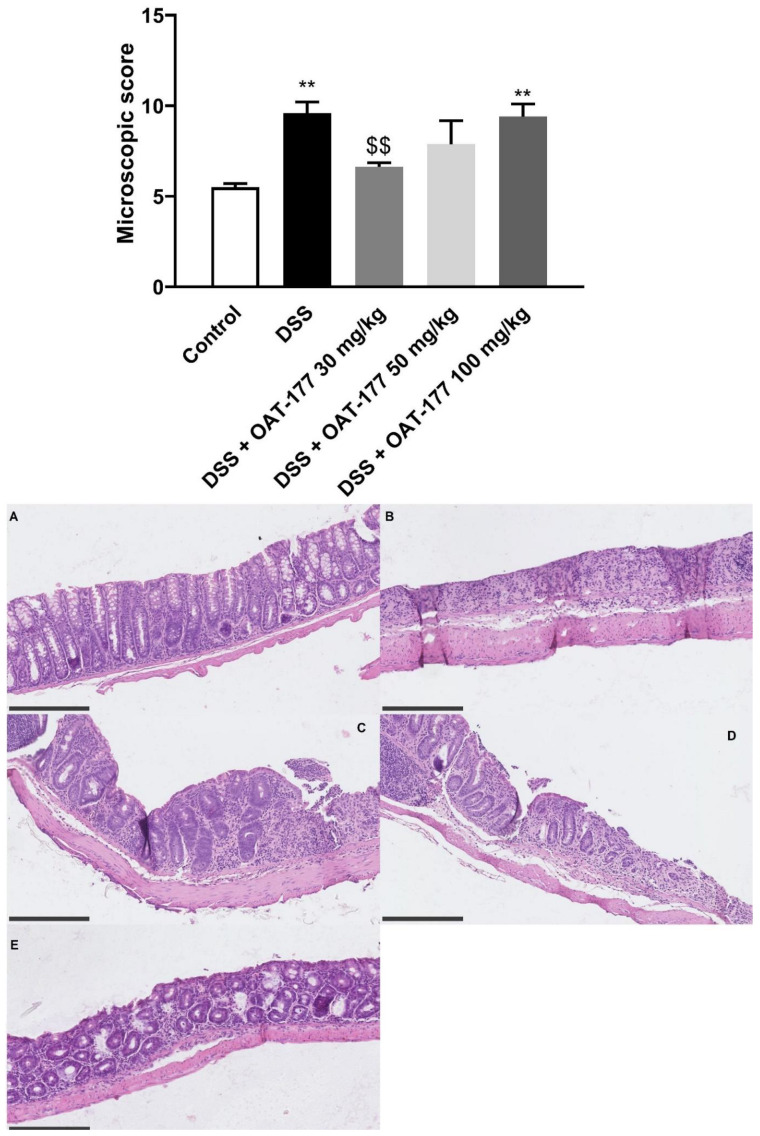
The effect of OAT-177 administered intragastrically in the short-term therapeutic protocol on the microscopic score in a dextran sulfate sodium (DSS)-induced colitis. Data are expressed as mean ± SEM. The statistical significance from the one-way ANOVA, Tukey’s post hoc test: ** *p* < 0.01 vs. control group; and $$ *p* < 0.05 vs. DSS-only treated group. Representative photos of hematoxylin and eosin staining of colon samples ((**A**): control group; (**B**): DSS-only treated group; (**C**): OAT-177 30 mg/kg; (**D**): OAT-177 50 mg/kg; and (**E**): OAT-177 100 mg/kg). Scale bar = 250 μm.

The parameters taken into consideration during microscopic evaluation included muscle thickness, cell infiltration, mucosal architecture, crypts morphology, and the presence of goblet cells. DSS treatment significantly increased the microscopic score (9.58 ± 0.62 for DSS-only treated animals vs. 5.5 ± 0.20 for control animals, *p* < 0.01). The lowest dose of OAT-177 (30 mg/kg, p.o.) caused a statistically significant decrease in the microscopic score (6.62 ± 0.23, *p* = 0.01) (Figure 2).

### 2.2. OAT-177 Administered Intragastrically in the Short-Term Therapeutic-Protocol-Induced Changes in the Inflammatory Cytokine Profile

**Figure 3:**
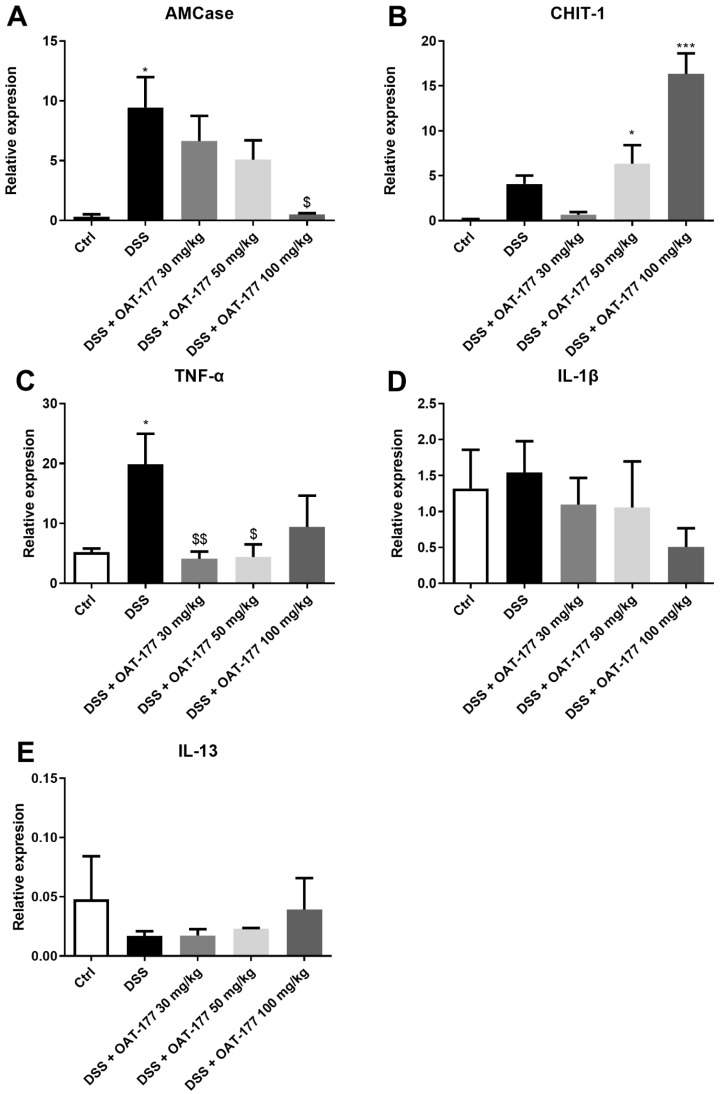
AMCase (**A**), Chitinase 1 (**B**), and selected cytokine: TNF-α (**C**), IL-1β (**D**), and IL-13 (**E**) mRNA expression in the mouse colon for control, DSS-only treated mice, and mice with DSS-induced colitis treated with OAT-177 at doses of 30, 50, and 100 mg/kg (p.o.) in the short-term therapeutic protocol. *** *p* < 0.001, * *p* < 0.05 vs. control group; $$ *p* < 0.01, $ *p* < 0.05 vs. DSS-only treated group.

The expression of genes for AMCase, CHIT-1, and selected cytokines (TNF-α, IL-1β, and IL-13) was assessed in the mouse colon samples at the mRNA level. OAT-177 at the dose of 100 mg/kg (p.o.) significantly inhibited the AMCase compared to DSS-only treated mice (0.52 ± 0.21 vs. 9.44 ± 7.17, Figure 3A). We also observed a reverse dose-dependent shift in the relative expression of chitinase 1: the group with DSS-induced colitis treated with OAT-177 at the dose of 100 mg/kg (p.o.) showed the highest chitinase 1 mRNA level (16.35 ± 2.28), while the expression in the group treated with the dose of 30 mg/kg (p.o.) was similar to the control animals (0.64 ± 0.94 vs. 0.13 ± 0.08, respectively) (Figure 3B). OAT-177 at doses of 30 and 50 mg/kg (p.o.) significantly decreased TNF-α mRNA levels compared to DSS-only treated mice (OAT-177 30 mg/kg: 4.11 ± 1.20; OAT-177 50 mg/kg: 4.40 ± 2.10; and DSS: 19.88 ± 5.07, Figure 3C). The IL-1β and IL-13 expression levels showed no significant changes between the groups (Figure 3D,E).

### 2.3. The Anti-Inflammatory Effect of OAT-177 Is Also Present after Intraperitoneal Administration in the Short-Term Therapeutic Protocol

**Figure 4:**
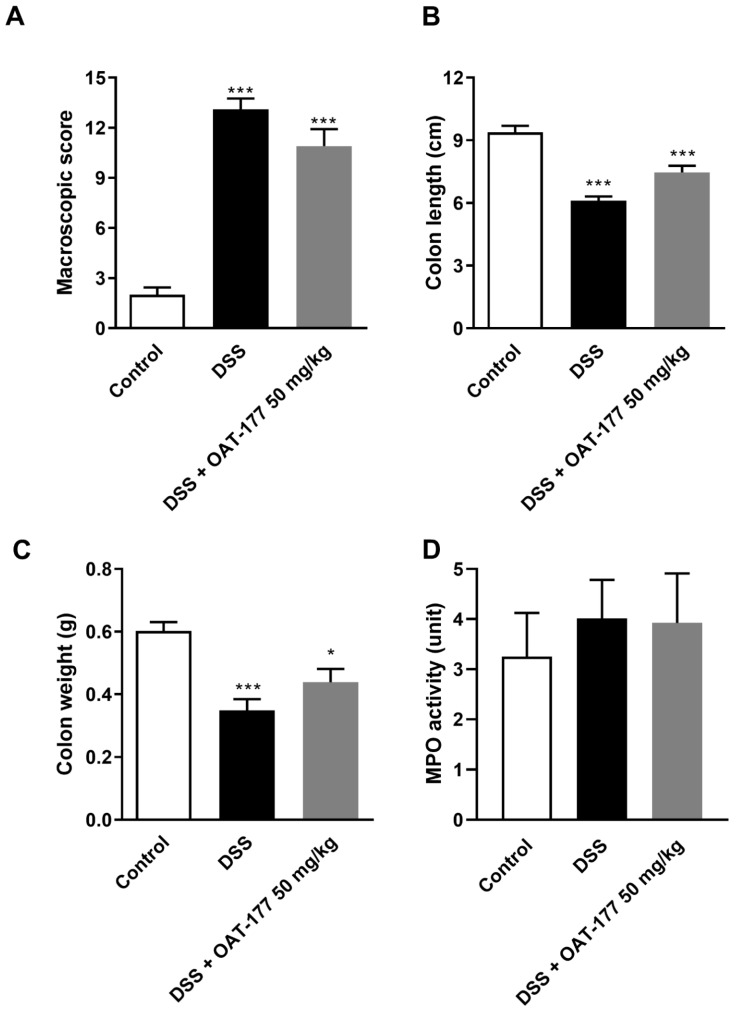
The effect of OAT-177 administered intraperitoneally in the short-term therapeutic protocol on: (**A**) macroscopic score, (**B**) colon length, (**C**) colon weight, and (**D**) myeloperoxidase (MPO) activity in a dextran sulfate sodium (DSS)-induced colitis. Data are expressed as mean ± SEM. Statistical significance from the one-way ANOVA, Tukey’s post hoc test: *** *p* < 0.001, * *p* < 0.05 vs. control group.

Based on the macroscopic score and colon weight, OAT-177 administered i.p. in a dose of 50 mg/kg did not reverse the effect of DSS-induced colitis (Figure 4A). However, i.p. administration of OAT-177 in the DSS-treated group significantly increased colon length compared with DSS-only treated mice (7.46 ± 0.32 cm vs. 6.1 ± 0.21 cm; *p* < 0.01) (Figure 4B) and non-significantly increased colon weight (Figure 4C). The MPO activity in this experiment did not present any significant differences between the groups (Figure 4D).

### 2.4. OAT-177 Administered Intragastrically in the Long-Term Therapeutic-Protocol-Attenuated Colitis in the DSS-Induced Mouse Model

**Figure 5:**
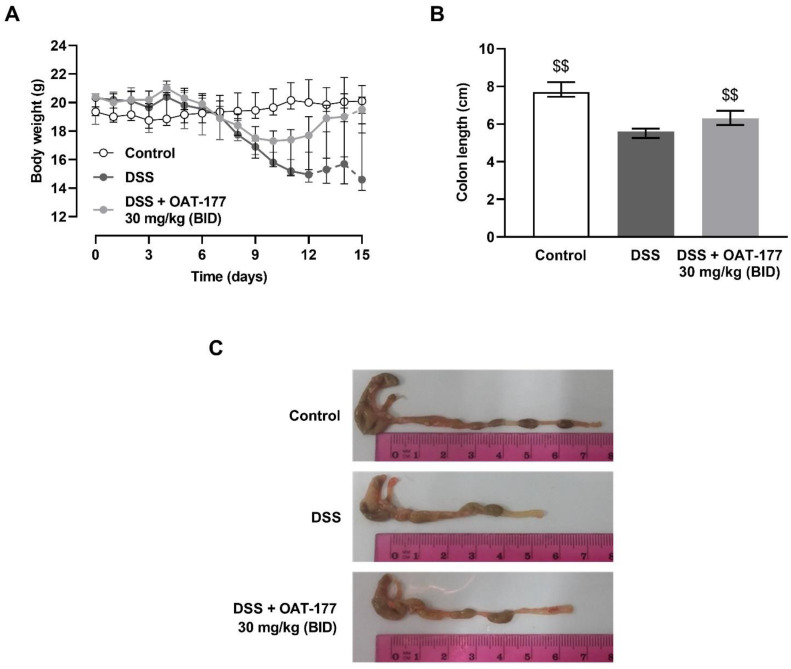
The effect of OAT-177 (30 mg/kg) administered intragastrically in the long-term therapeutic protocol on: (**A**) body weight changes (dotted line indicates the onset of mortality) and (**B**) colon length at day 15 in a dextran sulfate sodium (DSS)-induced colitis. Data are expressed as medians ± IQR. (**C**) Colons dissected with distal ileum and caecum (representatives for each group). Statistical significance from the U-Mann Whitney test: $$ *p* < 0.01 vs. DSS-only treated group.

To further determine the anti-inflammatory effect of the tested inhibitor, we administered OAT-177 (30 mg/kg, intragastrically, twice daily from day 3) in the long-term therapeutic protocol. The body weight (Figure 5A) and colon length at day 15 (Figure 5B) had significantly improved parameters compared to DSS-induced colitis.

## 3. Discussion

In this report, we demonstrate the efficacy of a selective murine AMCase inhibitor, compound OAT-177, in the DSS-induced mouse models of colitis. In the short-term therapeutic protocol, OAT-177 administered intragastrically in a dose of 30 mg/kg BID produced a significant anti-inflammatory effect and improved overall colon health. Importantly, we also found that at higher doses of the inhibitor, the expression of mAMCase is reversed to the level of a healthy control mouse. However, this is accompanied by a profound induction of a second chitin-cleaving enzyme—chitotriosidase-1, known to be a strongly pro-inflammatory protein. The net result of such a compensation mechanism is an exacerbation of the disease observed at higher doses of mAMCase inhibitor. Despite ongoing studies, the physiological functions of chitinases remain to be fully elucidated, but these enzymes have already been shown to be involved in the pathogenesis of various human inflammatory and fibrotic disorders [19,20,21]. In terms of IBD, particularly YKL-40 and, to somewhat less extent, AMCase appear to play a pivotal role [22,23]. The role of YKL-40 in IBD was recently summarized in a review published by our group [9]. Briefly, YKL-40 is actively produced by colonic epithelial cells, lamina propria macrophages, and neutrophils under inflammatory conditions of colon [24,25]. YKL-40 is also engaged in enhancing bacterial adhesion and invasion into colonic epithelial cells [22,26]. AMCase is highly expressed in the glandular cells of the stomach and intestinal tissues under normal physiological conditions, purportedly to aid in host defense and food processing. What is noteworthy, as revealed by Okawa et al., is that the expression level of AMCase and its chitinolytic activity in the stomach tissues is significantly higher in mice than in humans [27]. AMCase is also present in the mouse lung, although at a considerably lower level [27]. AMCase was demonstrated to be activated during the Th2-type inflammatory responses in OVA- and HDM-induced mouse models and asthma patients [11].

Consequently, inhibition of chitinases AMCase and CHIT1, and also of the chitinase-like protein YKL-40 (CHI3L1), may prove a viable strategy for treatment of inflammatory and fibrotic diseases, including colitis [21,28,29]. Previously, both selective and non-selective chitinase inhibitors were investigated principally in inflammatory and fibrotic models of pulmonary diseases. The first selective AMCase inhibitor bisdionin F [30] showed in vivo efficacy in the OVA-induced allergic airway inflammation mouse model by attenuated lung chitinase activity, reduced eosinophil influx, and improved ventilatory function. The Wyeth research group used a combination of high-throughput screening with in silico screening to identify an AMCase inhibitor (Wyeth-1 compound), which reduced AMCase enzymatic activity in the bronchoalveolar lavage fluid in allergen-challenged mice after oral dosing [31]. Further investigation of the aminotriazole series (analogs of the Wyeth-1 compound improved in terms of activity and pharmacokinetic properties) by Mazur et al. led to the discovery of highly potent chitinase inhibitors displaying various modes of AMCase vs. CHIT-1 selectivity. As mentioned above, a selective mAMCase inhibitor, compound OAT-177, showed a significant anti-inflammatory efficacy in acute house dust mite (HDM)-induced allergic airway inflammation [15].

The same group reported the discovery of the compound OAT-1441, which proved to be more than 130 times more active against human AMCase (IC_50_ = 7 nM) versus human CHIT-1 (IC_50_ = 915 nM) [32].

Also, dual AMCase and CHIT1 inhibitors, compound 30 described in reference [33] and OATD-01, proved efficacious in HDM-induced pulmonary inflammation and the bleomycin-induced pulmonary fibrosis mice model [28,33].

The possible application of chitinase inhibitors in the treatment of gastrointestinal inflammatory diseases has been far less advanced. Mizoguchi group demonstrated that naturally occurring chitinase inhibitors, methylxanthine derivatives including caffeine, theophylline, and pentoxifylline reduced inflammation in mice treated with DSS via CHI3L1 inhibition [14,34,35]. The downregulation of CHI3L1 expression was associated with reduction of the bacterial invasion effect. They also demonstrated that in IEC lines treated with caffeine the CHI3L1 mRNA expression is decreased and correlates with a reduction of bacterial invasion in a dose-dependent manner. Peterson et al. [36] showed that intra-rectal administration of pentoxifylline or 1-(5-hydroxyhexyl)-3,7-dimethylxanthine in a mouse 2,4,6-trinitrobenzenesulfonic acid (TNBS)-induced colitis model attenuated colonic inflammation and intestinal fibrosis. The same research group demonstrated similar efficacy of pentoxifylline in TNBS-induced colitis in rats [36]. Murthy et al. showed that a combined therapy of pentoxifylline and anti-TNF antibody in DSS-induced colitis mice can be beneficial to limit the side effects associated with anti-TNF antibody treatment alone [37]. Ex vivo studies also illustrated that treatment of pentoxifylline of peripheral mononuclear cells from the inflamed mucosa of CD and UC patients reduced TNF secretion by 50 %.

All the above prompted us to investigate whether the selective mAMCase inhibition would be effective in the DSS-induced mouse model of colitis. As detailed in the Results section, we found that oral administration of a 30 mg/kg dose of OAT-177 exerted the expected, significant anti-inflammatory effect in both the short-term and long-term therapeutic protocols. Our results also suggest that for the human acidic mammalian chitinase (hAMCase) inhibition to be an effective IBD treatment, it is important to ensure the local presence of the inhibitor in the colon. It would further implicate the appropriate hAMCase inhibitors design aimed at molecules that should be poorly absorbed from the gastrointestinal tract to allow them to act locally.

In the current study, a strong escalation of expression of mAMCase in the DSS-challenged mice as compared to the control group was observed. This increase was attenuated by administration of the mAMCase inhibitor in a dose-dependent manner achieving the healthy control level at the highest (100 mg/kg) dose studied. Interestingly, an opposite observation was made regarding the expression of mCHIT-1 enzyme, whose level in this group increased 16-fold versus the control group. These findings are in line with the results of Mizoguchi et al. [34], who discovered that oral caffeine (reported as a pan-family 18 chitinase inhibitor) administration, downregulates CHI3L1 and AMCase expression, but induces CHIT-1 expression.

In summary, the present study reports the therapeutic effect of the selective mAMCase inhibitor, compound OAT-177, in the DSS-induced colitis model. The statistically significant positive impact of the administration of the drug was observed for the lowest dose investigated, while in higher doses it proved not to be effective. This may be driven by the induction of mouse CHIT-1 protein, which, as we show, is intensely produced to compensate for the lack of AMCase activity. While the TNF-α level rose only slightly and, on the other hand, it is well known that CHIT-1 is upregulated in inflammatory conditions, we speculate that its overexpression is a root cause of an exacerbation of the inflammation in the two higher-dosed mice groups. Therefore, the proper dosing of the selective AMCase inhibitor may be a critical factor for balancing the efficacy of the treatment versus its safety. Also, given the apparent role of CHIT-1 in colon inflammation, selective CHIT-1 inhibition or simultaneous inhibition of both chitinases may provide efficient strategies for treatment of colitis. Beside all the known differences between expression levels and the roles of the two chitinases in mouse versus human, this fact would also have to be taken into account, should targeting AMCase be contemplated as a strategy to treat IBD in patients. Also, while the expression of AMCase in healthy humans is known to be negligible, its level in IBD patients has not been studied so far. Both the serum concentration and the level of the enzyme in the diseased tissue would be of interest when planning the potential application of human AMCase inhibitors in the clinic.

## 4. Materials and Methods

### 4.1. Experimental Animals

Male BALB/c mice were obtained from the Animal Facility of the University of Lodz, Poland; the animals used in the experiments weighed 20–25 g (approx. 8 weeks of age). Female C57BL/6 mice were obtained from the Charles River Laboratories (Germany); the animals used in the experiments weighed 18–21 g (approx. 10 weeks old). The animals were housed at a constant temperature (22–23 °C) and maintained under a 12 h light/dark cycle with free access to laboratory chow and tap water. Animal protocols were approved by the Local Ethics Committee for Animal Research at the Medical University of Lodz (Approval Nos. 2/ŁB123/2019 and 17/ŁB132/2019) or by the Second Warsaw Local Ethics Committee for Animal Research (Approval No. 46/2015). All efforts were made to minimize animal suffering and to reduce the number of animals used.

### 4.2. Induction of Colitis

In the short-term therapeutic protocol, BALB/c mice (*n* = 6–8 per experimental group) received 4 % DSS (MW 40 kDa, Biochemica, PanReac AppliChem, Darmstadt, Germany) in drinking water from day 0 to day 5. On days 6 and 7 the animals received drinking water alone. Mice were sacrificed by cervical dislocation on day 7 and colonic damage was evaluated.

In the long-term therapeutic protocol, C57BL/6 mice (*n* = 6–7 per experimental group) received 3 % DSS (MW 36–50 kDa, MP Biomedicals) in drinking water from day 0 to day 7, and then regular drinking water until day 15. Mice were sacrificed on day 15 and colonic damage was evaluated.

In both experimental protocols, control mice were given drinking water throughout the whole experiment. Animal body weight was monitored daily.

### 4.3. Pharmacological Treatment

In the short-term therapeutic protocol, OAT-177 was administered twice daily from day 3 to day 6 after colitis induction: intragastrically (p.o., 100 µL/mouse) at doses of 30, 50, and 100 mg/kg, and intraperitoneally (i.p., 100 µL/mouse) at the dose of 50 mg/kg. Control and DSS groups received 0.9 % NaCl alone. In the long-term therapeutic protocol, OAT-177 was administered intragastrically twice daily from day 3 until the end of the experiment (last dose in the afternoon on day 14). The control and DSS-treated groups received the vehicle alone (20 % solutol, 5 % dextrose in water).

### 4.4. Macroscopic Evaluation of Colonic Damage

For total macroscopic inflammation score evaluation, the colon was removed immediately after cervical dislocation and weighed (including fecal content). The colon was opened longitudinally and the feces removed. The well-established semiquantitative scoring system used throughout the study took into consideration the following parameters: colon length and weight scores (0–4), stool consistence (0–3), and colon epithelial damage, i.e., the number of ulcers (0–3), where score = 0 means no inflammation. The presence (score = 1) or absence (score = 0) of fecal blood was also recorded. The macroscopic scoring was performed in a blind manner.

### 4.5. Microscopic Inflammation Score Evaluation

Distal colon sections (approx. 0.5 cm in length) were stapled flat, mucosal side up, onto cardboard and fixed in 10 % neutral-buffered formalin for at least 24 h at 4 °C. Next, samples were dehydrated in sucrose and embedded in paraffin, sectioned at 5 μm and mounted onto slides. The hematoxylin-eosin-stained sections were examined using a Zeiss Axio Imager setup (Jena, Germany). The microscopic total damage score was determined based on the following parameters: presence (score = 1) or absence (score = 0) of goblet cell depletion, the presence (score = 1) or absence (score = 0) of crypt abscesses, the destruction of mucosal architecture (normal = 1, moderate = 2, extensive = 3), the extent of muscle thickening (normal = 1, moderate = 2, extensive = 3), and the presence and degree of immune cell infiltration (normal = 1, moderate = 2, transmural = 3). The microscopic scoring was performed in a blind manner.

### 4.6. Determination of Tissue Myeloperoxidase Activity

To monitor the degree of inflammation, myeloperoxidase (MPO) activity was determined using a standardized method, as described earlier [17]. Briefly, colon segments (weighing approx. 30 mg) were homogenized in a hexadecyltrimethylammonium bromide (HTAB) buffer (0.5 % HTAB in 50 mM potassium phosphate buffer, pH 6.0; 50 mg tissue/mL). Homogenates were centrifuged (15 min, 13,200 × *g*, 4 °C). On a 96-well plate, 200 μL of 50 mM potassium phosphate buffer (pH 6.0), supplemented with 0.167 mg/mL of O-dianisidine hydrochloride and 0.05 μL of 1 % hydrogen peroxide, was added to 7 μL of the supernatant. Absorbance was measured at 450 nm after 30 and 60 s (iMARK Microplate Reader, Biorad, Hertfordshire, UK). All measurements were performed in triplicate. MPO activity was expressed in milliunits per gram of wet tissue, 1 unit being the quantity of enzyme able to convert 1 μmol of hydrogen peroxide to water in 1 min at room temperature.

### 4.7. RNA Isolation

Total RNA was isolated from snap-frozen colonic tissue obtained from the control and experimental groups using a Total RNA Mini Plus kit (A&A Biotechnology, Gdansk, Poland). RNA was eluted from ion-exchange columns by DEPC water (50 μL). The quality and quantity of RNA samples were determined with a Colibri Microvolume Spectrophotometer (Biocompare, San Francisco, CA, USA). The RNA was characterized with an A260 nm/A280 nm ratio, which was in the range of 1.7–2.0.

### 4.8. Reverse Transcription and Real-Time RT PCR

Total RNA (2 μg) was transcribed with a cDNA using High-Capacity cDNA Reverse Transcriptase Kit (Life Technologies, Carlsbad, CA, USA). Quantitative analysis was performed using fluorescently labeled TaqMan probes: Mm01291360_m1 for chitinase 1, Mm00458221_m1 for AMCase, Mm00443258_m1 for TNF-α, Mm00434228_m1 for IL-1β, Mm00434204_m1 for IL-13, and Mm99999915_g1 for GAPDH as endogenous control (Life Technologies, Carlsbad, CA, USA), and a TaqMan Gene Expression Master Mix without UNG (Life Technologies, Carlsbad, CA, USA) in accordance with manufacturer’s protocol on a Lightcycler 96 apparatus (Roche, Warsaw, Poland). The cycle parameters were designed as follows: initial denaturation at 95 °C for 10 min, 55 cycles of sequential incubations at 95 °C for 15 s, and at 60 °C for 1 min. All experiments were performed in triplicate and the obtained results were normalized to the expression of GAPDH. The fluorescent dye emission was a function of the cycle number. The Ct value corresponded to the threshold cycle number at which PCR amplification reached a significant threshold. The relative expression level was calculated as 2−∆Ct × 1000 and the results are expressed as the number of examined mRNA copies per 1000 copies of mRNA for GAPDH.

### 4.9. Statistical Analysis

The statistical analysis was performed in a Prism 9 (GraphPad Software Inc., La Jolla, CA, USA) with the use of one-way ANOVA followed by Tukey’s post hoc test. For non-parametric analysis, the U Mann-Whitney test was used. The data with normal distribution were presented as mean with standard error of the mean (SEM); otherwise, median with interquartile range (IQR) was shown. Differences at *p* < 0.05 were considered statistically significant.

## Data Availability

The data presented in this study are available on request from the corresponding authors.
